# Ion content, antioxidant enzyme activity and transcriptional response under salt stress and recovery condition in the halophyte grass *Aeluropus littoralis*

**DOI:** 10.1186/s13104-022-06090-4

**Published:** 2022-06-11

**Authors:** Seyyed Hamidreza Hashemipetroudi, Gholamreza Ahmadian, Farzaneh Fatemi, Ghorbanali Nematzadeh, Ahad Yamchi, Markus Kuhlmann

**Affiliations:** 1 Genetics and Agricultural Biotechnology Institute of Tabarestan (GABIT), Sari Agricultural Sciences and Natural Resources University, PO Box 578, Sari, Iran; 2 National Institute of Genetic Engineering and Biotechnology (NIGEB), Tehran, Iran; 3 Department of Plant Breeding and Biotechnology, Gorgan University of Agricultural Sciences & Natural Resources, Gorgan, Iran; 4 RG Heterosis, Leibniz Institute of Plant Genetics and Crop Plant Research (IPK), Gatersleben, Germany

**Keywords:** Halophyte, Salt stresses, Catalase, Superoxide dismutase, Ascorbate peroxidase, RT-qPCR, Elemental analysis

## Abstract

**Objective:**

In contrast to glycophytes, halophyte plants have evolved unique morphological and physiological mechanisms to deal with abiotic stress. This study presents the physiological responses of *Aeluropus littoralis*, a halophyte grass, to salt stress and recovery conditions on the molecular level.

**Results:**

Elemental analysis showed that Na^+^ concentration increased in the analyzed tissue during salt stress application, and declined at recovery condition. With the exception of root tissue, comparable trends of K^+^, Ca^2+^, and Mg^2+^ concentrations were observed (decreased during salt stress, increased during recovery). Salinity led to an increase in total chlorophyll (Chl), Chl a, and carotenoids content, while Chl b content decreased. The level of the proline amino acid associated with drought and salt stress was increased. Here APX, POD, and SOD activity were strongly detectable in roots and reduced later under recovery conditions. RT-qPCR revealed up-regulation of antioxidant genes at S1 and S3 in the root but down-regulation in recovery conditions. This study found a significant halophyte index for understanding the processes of salinity tolerance in *A. littoralis*. These findings may provide insight into the role of antioxidant enzymes during salt stress and the mechanism underlying the plant's response to stress.

**Supplementary Information:**

The online version contains supplementary material available at 10.1186/s13104-022-06090-4.

## Introduction

In modern agriculture, salt stress is one of the most important abiotic stressors that threatens crop growth and development [1]. Due to increasing global demand for food, agricultural expansion to salt-affected areas is inevitable [2]. Therefore, the development of salinity tolerant crops is crucial for global food security [3, 4]. To achieve this goal, it is necessary to understand the effects of high salinity on plant morphology, physiological processes, biochemical and metabolic responses, as well as gene expression [5, 6].

Halophytes represent an interesting example of a stress-tolerant plant [7, 8]. Halophytic plants have evolved a diverse set of adaptation strategies and tolerance mechanisms to survive in most extreme saline habitats [1, 9]. Crop improvement in terms of salinity and drought tolerance necessitates the application of halophyte research findings [10, 11]. The genetic potential of halophytic *Poaceae* can be used to improve glycophyte crops by identifying novel salt-responsive promoters and/or genes based on these resources [12–14].

In order to improve the knowledge on the adaptability of halophytes to salt stress, the physiological and molecular response of *Aeluropus littoralis* as a monocotyledonous halophyte model was evaluated under salt stress (600 mM NaCl treatment) and recovery conditions. Traits related to photosynthesis (Chl a, Chl b, and the total Cars), ion contents (Na^+^, K^+^, Ca^2+^, and Mg^2+^), and antioxidant enzyme activity (SOD, CAT, POD, and APX) were investigated in this study. On the molecular level, the mRNA abundance of *AlSOD*, *AlCAT,* and *AlAPX* genes at different time-points of salt stress and recovery conditions was analyzed and described.

## Main text

### Methods

#### Plant material and treatments

To ensure the uniformity of plants in this study, the cuttings of one mother plant of *A. littoralis* were collected from Isfahan province (32° 33′ 03.5"N 52° 29′ 31.3"E) were used in this study. *Aeluropus* plants were identified by Dr. Seyed Hassan Zali, and A specimen of the analyzed plants was deposited at the herbarium GAT under voucher number 70486. The growth conditions of *Aeluropus* seedlings were adjusted according to Fatemi et al. [7]*.* (Additional file 1: Fig. S1). At the end of the sixth passage, the shoot and root samples were collected at 6 h (S1), 48 h (S2), and after one week (S3). The salt-treated plants were transferred to a sodium chloride-free Hoagland's solution and further cultivated, designated as a recovery condition. The samples for recovery conditions were collected after 6 h (R1), 48 h (R2), and one week (R3) (Additional file 2: Fig. S2).

#### Physiological, Ion content and antioxidant enzyme activity measurement

Chlorophyll (Chl) a, Chl b, and the total carotenoids (Cars) were extracted from aerial tissue using methanol, and the absorbance of the extract was measured at 665.2, 652.4, and 470 nm [15] using the Biochrom WPA Biowave II spectrophotometer. For elemental analysis, plant material was dried at 65 °C for 48 h, and its dry weight was recorded. Samples (three biological replications) were digested in 70% nitric acid and 30% hydrogen peroxide for 2.5 h at 120 °C, and then the elements of Na^+^, K^+^, Ca^2+^, and Mg^2+^were measured by ICP-OES.

Total protein and ROS scavenging enzymes were purified from root and leaf samples according to Kaur et al. 2016 procedures [16]. Total soluble proteins were measured by the Bradford assay. The activity of SOD (EC 1.15.1.1) was measured using the reduction of nitro blue tetrazolium (NBT) [17]. CAT (EC 1.11.1.6) activity was assayed based on the decomposition of H_2_O_2_ [18]. The APX (EC 1.11.1.11) was assayed according to the method described by Nakano and Asada [19]. Assays of POD (EC 1.11.1.7) activity were carried out using guaiacol as the hydrogen donor [20].

#### RT-qPCR

Total RNAs were extracted using Threezol reagent (Riragene company, Iran). The quality and quantity of RNA samples were estimated by Nanodrop spectrophotometer (Biochrom WPA Biowave II, UK). The sequences of *CAT*, *SOD, cAPX,* and *pAPX* as target genes and *actin* as a reference gene were obtained from the nucleotide database at NCBI. Primers of target and reference genes (except for *APXs*) were designed using Primer 3 software [21]. The gene-specific primers for two isoforms of cytosolic and peroxisomal *APX* were designed with the AlleleID v7.0 software (Premier, CA). A taxa-specific/cross-species assay was conducted with aligned sequences of various *APX*, which could detect *cAPX* or *pAPX* only (Table 1). The RT-qPCR was accomplished in the CFX96 real-time PCR instrument (Bio-Rad) according to Hashemi et al. [22, 23]. The specificity of primers was checked by melt curve analysis (Additional file 3: Fig. S3). RT-qPCR data were analyzed according to Livak and Schmittgen method [24].

**Table 1:** The list of primer and their features was used in this study

abbreviation	Gene name	Accession no.	Primer sequence	Amplicon length (bp)
*AlCAT*	Catalase	HQ389206.1	CAACTTCCCCGTCTTCTTCA TGCGACAGAAAGTCGAACAC	119
*AlSOD*	copper/zinc superoxide dismutase	HM107007.2	CAAATGGCTGCATGTCAACT TGCTCCAGCTGTCACATTTC	113
*AlpAPX*	Peroxisomal ascorbate peroxidase	JF907687.1	ACGATGCTGGAACTTACGA GGCTGTGCTCTTCCTCAA	78
*AlcAPX*	Cytosolic ascorbate peroxidase	JF819725.1	CTCCTACGCCGACCTCTA CATCTGCTTGACGAAGACTTG	175
*AlUBQ2*	60S ribosomal protein L40-1	EE594598	CTTGGTCTGCTGTTGTCTTG CACGGTTCACTTATCCATCAC	200
*AlRPS3*	Ribosomal protein S3 family protein	JZ191044.1	ATTCACTGGCTGACCGGATG GTGCCAAGGGTTGTGAGGTC	107

## Results and discussion

### Changes in chlorophyll and carotenoids content

**Fig. 1:**
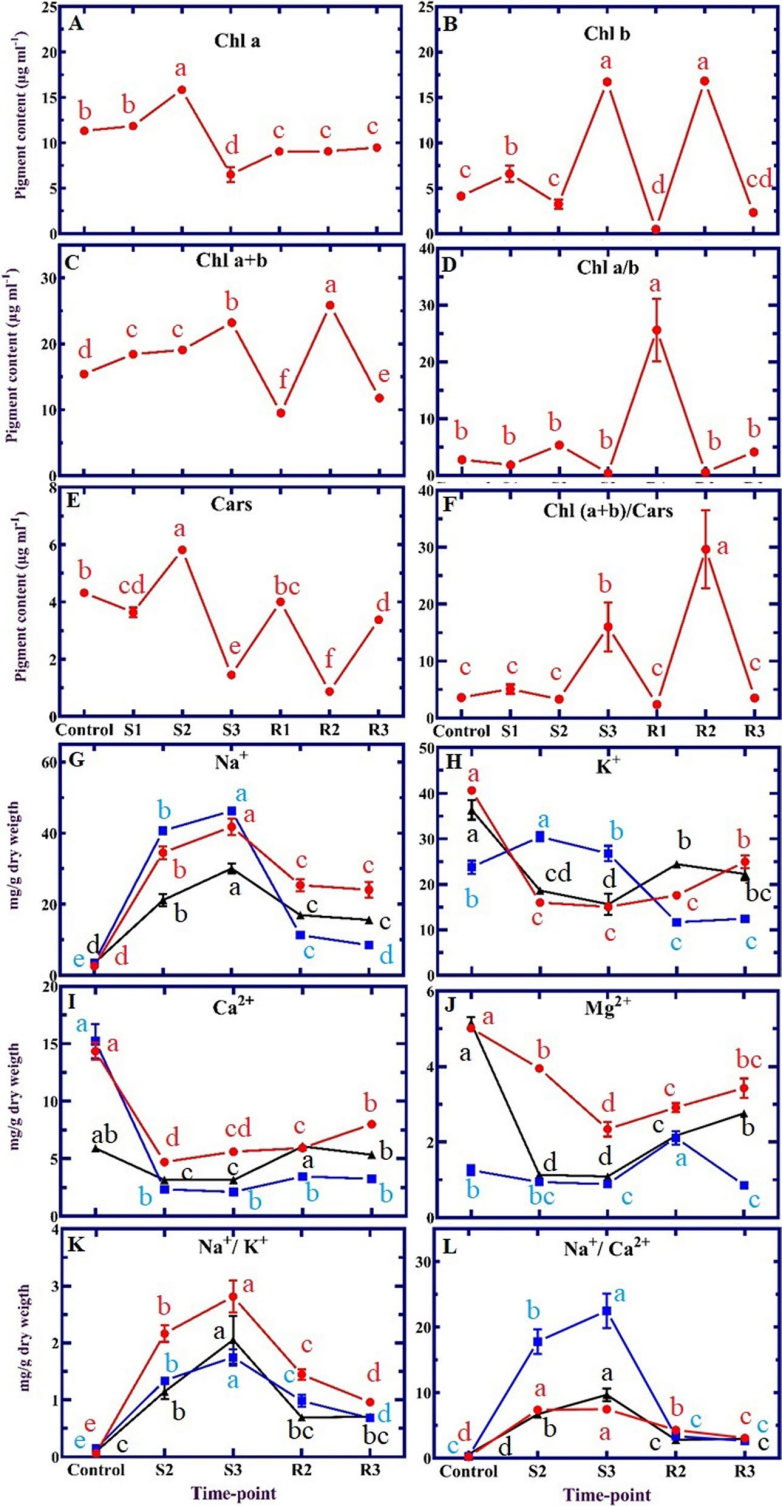
Different physiological characteristics and elemental parameters of *Aeluropus littoralis* were trended during salinity stress (600 mM NaCl) and recovery. **A** chlorophyll a (Chl a), **B** chlorophyll b (Chl b), **C** total chlorophyll (Chl (a + b)), **D** chlorophyll a to chlorophyll b ratio (Chl a/b), **E** carotenoids (Cars), **F** total chlorophyll to carotenoids ratio (Chl (a + b)/Cars), **G** Sodium (Na^+^), **H** potassium (K^+^), **I** calcium (Ca^2+^), **J** magnesium (Mg2+), **K** sodium to potassium ratio (Na^+^/K^+^), **L** sodium to calcium ratio (Na^+^/Ca^2+^) content. The trend in leaf, root and stem tissues was illustrated by the red, blue and black lines, respectively. The values represent the mean (± SE) of three biological replicates. According to Duncan's multiple test, the treatments exhibited a statistically significant difference at the 5% level and were marked with distinct letters

The content of Chl a increased sharply at 48 h during the salt stress treatment and then decreased significantly at the end of the salt stress treatments (Fig. 1A). Chl abundance during the recovery condition tends to return to control levels without reaching this initial value. In contrast, a significant increase in Chl b content (Fig. 1B) and subsequent Chl (a+b)/Cars ratio (Fig. 1F) were observed at S3 and R2 time-points (p-value < 0.01). A significant difference was observed in the Chl a/Chl b ratio between the R1 time-point and others (Fig. 1D).

The Cars quantity showed a strong dynamic response to the stress conditions (Fig. 1E). The Chl (a+b) content increased over the entire stress treatment compared to control (Fig. 1C). Chl and Cars as a key photosynthetic pigments perform vital physiological functions and photosynthetic processes in plants [25]. A recent literature review shows that the effect of salinity stress on photosynthetic pigments is highly complex and plant-specific [26]. In this study, the levels of light-harvesting pigments (Chl) were not equally sensitive to salt stress and recovery conditions (Fig. 1). The reduction of Chl a abundance one week after the salt stress could be attributed to the inhibition of chlorophyll biosynthesis and/or the stimulation of chlorophyll degradation [27]. On the other hand, plants under salt stress (S3) and recovery condition (R2) had more Chl b content than Chl a. Our finding is in agreement with the described inter-conversion of Chl a and Chl b in the chlorophyll cycle, which occurs via 7-hydroxymethyl chlorophyll [28]. In general, understanding the optimization of leaf function under salt stress condition requires more investment in leaf resources in light-harvesting than energy processing. Our finding might indicate the optimization of the halophytes to withstand salinity stress. Contrary to several previous findings [29, 30], the present study showed that the levels of Chl a and Chl b (except R2) were not restored in stressed plants following recovery.

### ICP–OES analysis

The most important factor here is the detection of sodium ions, as they reflect the amount of salt taken up from the soil. As *A. littoralis* is able to secrete salt through its salt glands, the level of detected sodium ions is a proxy for the level of in vivo salt stress in the respective tissue. Na^+^ levels in salt-stressed plants increased in the S2 and S3 time-points and decreased in the R2 and R3 conditions (Fig. 1G). The most variation in Na^+^ concentration was observed in root tissue by the highest and lowest levels of Na^+^ in salt stress and recovery conditions, respectively. In contrast, K^+^ accumulation in leaves and stems decreased during salt stress and increased during recovery (Fig. 1H). An increase in the ratio of Na^+^/K^+^ was observed in S2 and S3, while the decreasing trend was seen in recovery conditions (Fig. 1K). Ca^2+^ content significantly declined in leaf, root, and stem during salt stress compared to control, whereas it increased significantly in leaf and stem during the recovery condition (Fig. 1I). The trend of the Na^+^/Ca^2+^ ratio was similar to the Na^+^/K^+^ ratio with a minor difference (Fig. 1L). Mg^2^^+^ concentration gradually decreased during salt stress and gradually increased after transferring to recovery conditions (Fig. 1J).

High concentrations of Na^+^ decrease the amount of available K^+^, Mg^2+^ and Ca^2+^ by displacing membrane-bound Ca^2+^ [31]. Furthermore, Na^+^ may interfere with K^+^'s function as a cofactor, resulting in a direct toxic effect [32]. In our experiments, the absorption of Na^+^ and K^+^ seemed to be competitive processes. The Na^+^ concentrations increased mainly in stress conditions, while K^+^ concentrations were reduced, especially in the roots and stem. This phenomenon occurred because halophytes needed fewer K^+^ than glycophytes for growth [33], confirming the capability to substitute K^+^ with Na^+^ and finally salt tolerance. This is also the case that was reported by Belkheiri and Mulas (2013) in *Atriplex halimus* [34]. Even though salt stress causes damage to plants, the post-stress response is vital for recovery and survival. A previous report has described the recovery conditions allowed for the removal of salt-induced ion toxicity by reducing Na^+^ accumulation and increasing K^+^, Ca^2+^, and Mg^2+^ concentrations [29]. Other studies have found a decrease in oxidative damage and harmful ion accumulations [35].

### Proline, total protein and ROS enzyme activities

**Fig. 2:**
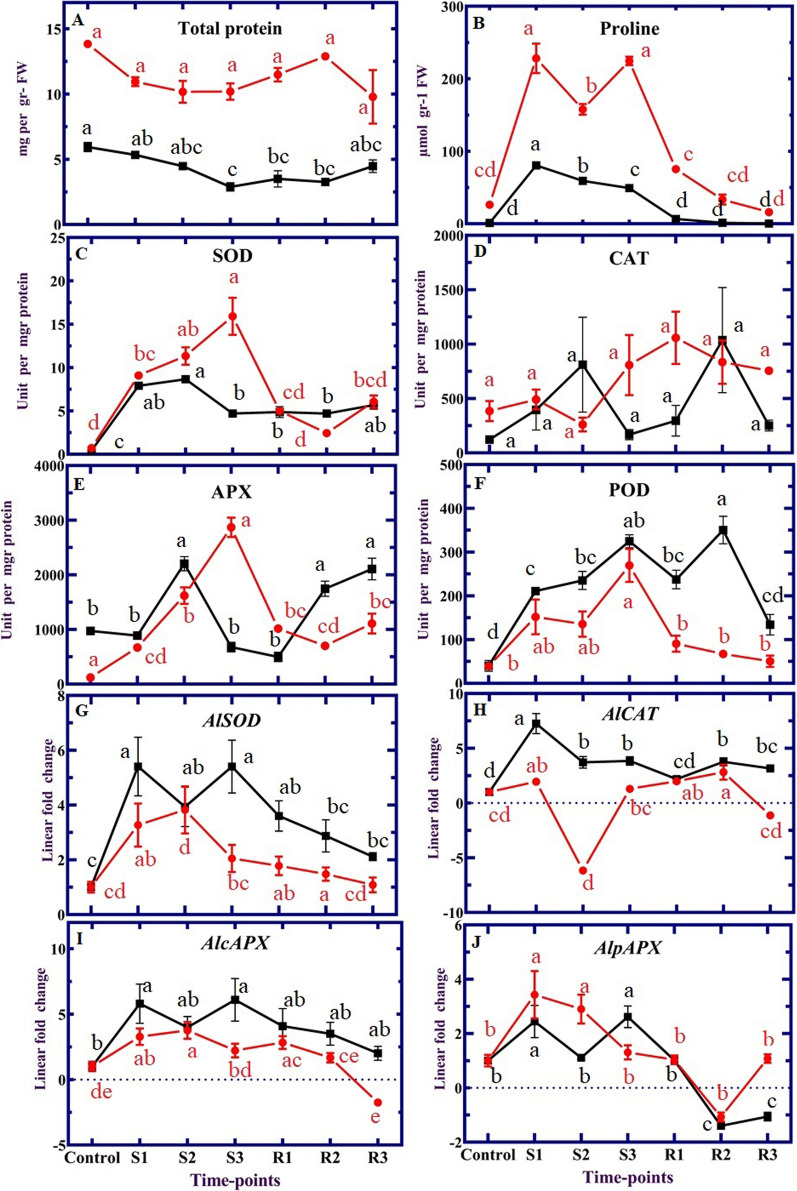
Trend of several antioxidant enzyme activities and their transcriptional responses under salinity stress and recovery. **A** total protein, **B** proline content, **C** superoxide dismutase (SOD), **D** activity of catalase (CAT), **E** ascorbate peroxidase (APX), **F** peroxidase (POD), **G** *AlSOD*, **H** *AlCAT*, **I** *AlcAPX*, and **J** *AlpAPX* in leaf and root of *A. littoralis*. The trend in leaf, root and stem tissues was illustrated by the red and black lines, respectively. The values represent the mean (± SE) of three biological replicates. According to Duncan's multiple test, the treatments exhibited a statistically significant difference at the 5% level and were marked with distinct letters

Total protein levels in salt-stressed plants and recovery conditions were significantly lower than in controls in both leaves and roots (Fig. 2A). In our study, proline content increased during salt treatment. Proline accumulation was described as its adaptive advantage under stress conditions [36]. Proline content was significantly increased during salt stress, but decreased in recovery conditions (Fig. 2B). Antioxidant enzyme activity also tended to increase in salt stress during different time-points, but decreased in those recovery conditions (Fig. 2C–F). These changes in proline and activity of antioxidant enzymes suggest that both are affected by salt stress conditions and might play an important role in adaptation to salinity-induced oxidative stress. As described in other studies, antioxidant enzyme related genes respond to salt stress treatment [37]. The CAT activity was higher in the recovery condition than in control and stress conditions in the leaf (Fig. 2D). SOD activity increased in both leaves and roots compared to the control (Fig. 2C). Stress conditions caused a profound increase in SOD activity to its maximum level at the S3 time-point, but it suddenly reduced in recovery condition in the leaf. The response of investigated enzymes was variable at different times of salinity and recovery conditions.

ROS are known as cytotoxic compounds that have important roles in homeostasis and cell signaling, which activates antioxidant defense mechanisms [38, 39]. There was a difference between CAT and SOD activity with salinity increasing and duration. The different changes for CAT and SOD activity indicated the importance of SOD rather than CAT in oxidative damage as the stress-time duration extended. The increasing changes of APX activity in the leaf during stress conditions showed that the highest APX activity occurred at the S3 time-point (Fig. 2E). POD activity increased significantly in stressed plants up to the highest level at the S3 time-point, while it decreased in the recovery condition in both leaf and root (Fig. 2F). The activities of SOD and POD in leaves showed similar trends. The decreases in CAT, APX and SOD activities could be attributed to ROS accumulation to toxic levels [40].

### RT-qPCR analysis

The *AlSOD* and *AlCAT* gene expressed similarly in leaf and root tissues. (Fig. 2G, H). The transcript levels of *AlCAT* in the leaves were consistent with CAT activities at different time-points, suggesting that CAT might be the main H_2_O_2_-scavenging enzyme to keep the balance of redox reactions in *A. littoralis*. The pattern of *AlcAPX* transcript level was highly similar to that of *AlSOD* mRNA, both in leaves and roots (Fig. 2I). The pattern of *AlpAPX* transcript levels in roots and leaves indicated an increase during salt application and a strong reduction during the recovery phase (Fig. 2J). *APX* isoforms of plants are found in chloroplasts, mitochondria, peroxisomes, cytosols and apoplasts [41]. The changes in transcript levels of *AlAPX* were not consistent with the APX activity, neither in leaf nor root, under stress and recovery conditions. From these results, the expressions of *AlAPX* and *AlSOD* were suppressed during the salinity-caused accumulation of H_2_O_2_. Similar results have been reported for rice [42]. This indicates a similar regulation via a common regulatory hub or transcription factor. Overexpression of antioxidant genes such as *SOD*, *APX*, and *CAT* was reported in wheat [43], Arabidopsis [44] and oat [45]. Our knowledge of the physiological mechanisms behind plants' responses to recovery is inadequate at this time. As evidenced by changes in chlorophyll fluorescence and oxidative stress indices, *Aeluropus* plants might perceive the recovery phase as a new stress condition.

## Conclusion

In conclusion, rising soil salinity is one of the most significant problems to agricultural productivity worldwide [46, 47]. However, the severity and period of stress have a significant impact on the composition and quantities of leaf photosynthetic pigments, elemental, and antioxidant enzymes, further research is needed to have a better knowledge of how salinity stress affects different species [48]. Moreover, a precise assessment of these traits would allow for the presentation of plant health as well as an indirect reflection of stress response. Findings of this study point to some specific characteristics of *A. littoralis* in response to ROS accumulation and oxidative stress. These results demonstrated important indices of halophyte describing salinity tolerance mechanisms in *A. littoralis*.

## Limitations

Due to missing *A. littoralis* complete genomic data, only small number of genes were selected for RT-qPCR expression analyses.

## Supplementary Information

**Additional file 1: Fig. S1**. The salt stress and recovery condition of *A. littoralis* and their growth parameters.

**Additional file 2: Fig. S2.** Experiment timeline.

**Additional file 3: Fig. S3.** Checking the primers specificity by melt curve analysis.

**Additional file 4: Fig. S4.** A leaf surface of *A. littoralis* plants grown at different watering regimes (left: Hoagland solution, and right: On the leaves of a stressed plant, salt gland secretions were visible in the form of salt crystals.

## Data Availability

A specimen of the analyzed plants was deposited by Dr. Markus kuhlmann at the Gatersleben herbarium (GAT) of the IPK under voucher number 70486. The datasets measured and analyzed during the study are available from the corresponding author upon reasonable request.

## Abbreviations

ROS: Reactive oxygen species
Chl: Chlorophyll
K^+^: Potassium
Ca^2+^: Calcium
Mg^2+^: Magnesium
CAT: Catalase
SOD: Superoxide dismutase
APX: Ascorbate peroxidase
O_2_^•−^: Superoxide
H_2_O_2_: Hydrogen peroxide
HO•: Hydroxyl radicals
1O_2_: Singlet oxygen
Cars: Carotenoids
RT-qPCR: Reverse transcription–qPCR
NCBI: National center for biotechnology information
ICP-OES: Coupled plasma optical emission spectrometer
