# Supplementary figures and images for: Ion content, antioxidant enzyme activity and transcriptional response under salt stress and recovery condition in the halophyte grass Aeluropus littoralis
Source: BMC Res Notes. 2022 Jun 11;15:201. doi: 10.1186/s13104-022-06090-4 (PMC9188045; doi:10.1186/s13104-022-06090-4)

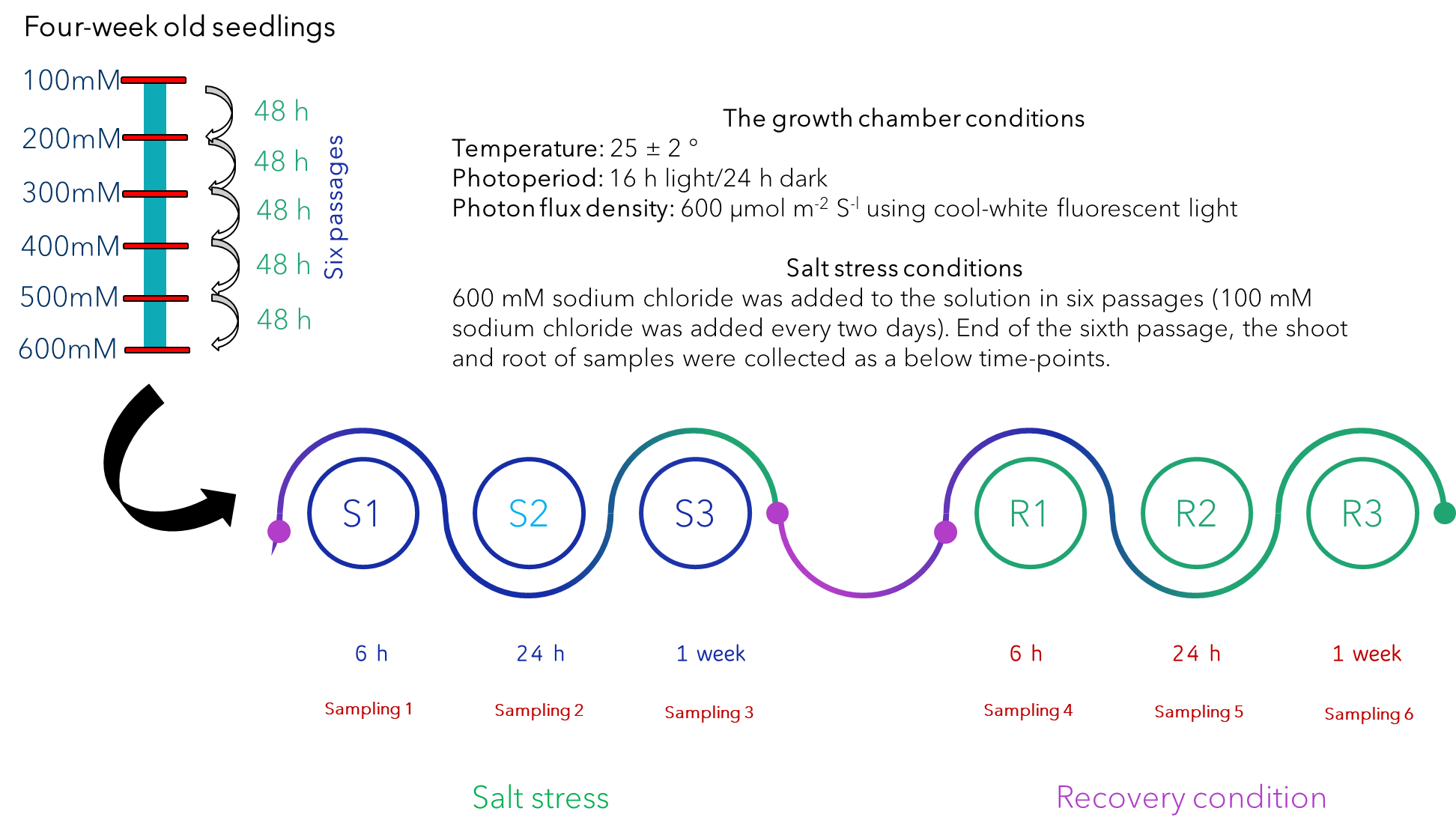

Supplement: Supplementary file 1 — Additional file 1: Fig. S1. The salt stress and recovery condition of A. littoralis and their growth parameters. [file 13104_2022_6090_MOESM1_ESM.docx]

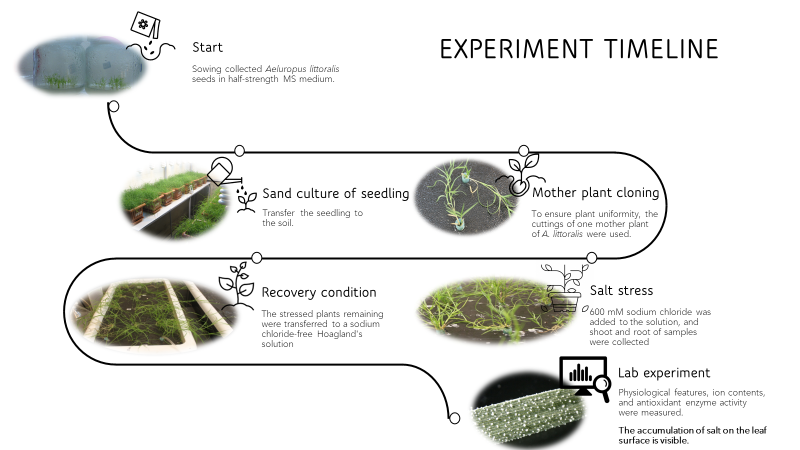

Supplement: Supplementary file 2 — Additional file 2: Fig. S2. Experiment timeline. [file 13104_2022_6090_MOESM2_ESM.docx]

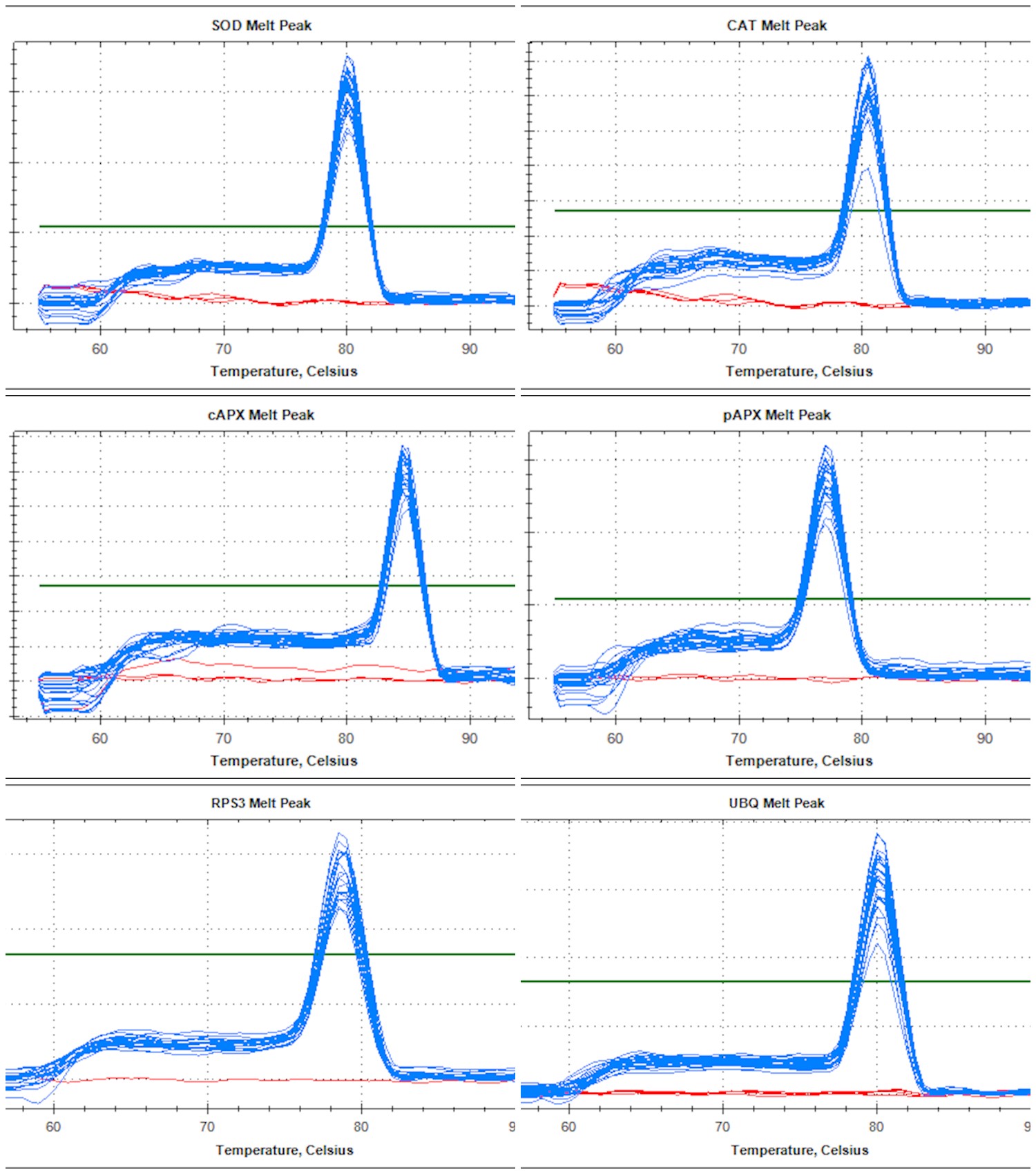

Supplement: Supplementary file 3 — Additional file 3: Fig. S3. Checking the primers specificity by melt curve analysis. [file 13104_2022_6090_MOESM3_ESM.jpg]

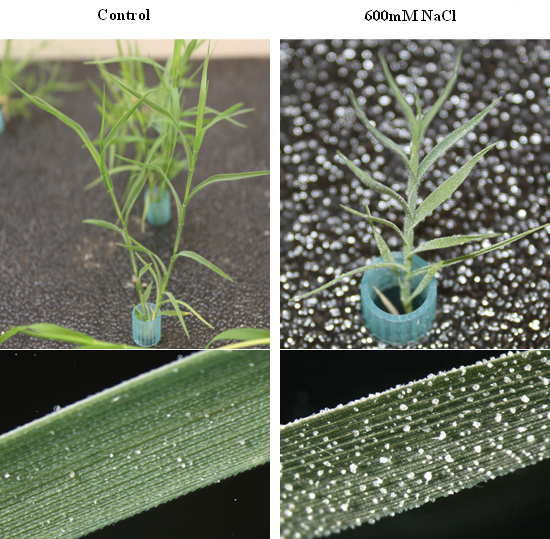

Supplement: Supplementary file 4 — Additional file 4: Fig. S4. A leaf surface of A. littoralis plants grown at different watering regimes (left: Hoagland solution, and right: On the leaves of a stressed plant, salt gland secretions were visible in the form of salt crystals. [file 13104_2022_6090_MOESM4_ESM.docx]
